# Hormonal contraceptive effects on the brain: considering the dual impact of endogenous and exogenous hormone flux

**DOI:** 10.1038/s41386-025-02267-0

**Published:** 2025-10-16

**Authors:** Ashley Ross, Tory A. Eisenlohr-Moul

**Affiliations:** 1 Medical Scientist Training Program, University of Illinois at Chicago, Chicago, IL USA; 2 Department of Psychiatry, University of Illinois at Chicago, Chicago, IL USA

**Keywords:** Neuroscience, Psychiatric disorders

Hormonal contraceptives (HCs), including combined estrogen-progestin oral contraceptives (COCs), are used by millions worldwide for reasons extending beyond contraception, such as managing acne, endometriosis, and premenstrual dysphoric disorder (PMDD). While research confirms that both endogenous ovarian hormones (E2, P4) and synthetic hormones in HCs affect neural structure and function [1], their impacts on affect, behavior, and cognition are notoriously inconsistent [2, 3]. This variability aligns with our emerging framework of dimensional hormone sensitivity [4], which posits that individuals differ not only in if they are behaviorally sensitive to hormones, but also to which specific changes they are sensitive (e.g., P4 metabolite surges, E2 withdrawal). Applying a dimensional framework to HC neuroscience [1–3] requires treating the vast pharmacological heterogeneity of HCs as potential mechanistic variables instead of a methodological nuisance. Each formulation creates a unique hormonal environment with respect to both exogenous and endogenous hormones, defining a set of hormonal triggers to which a susceptible person may react.

In this issue of *Neuropsychopharmacology*, Davignon et al. [5] examine relationships between COC use and fear regulation—a core feature of anxiety- and trauma-related disorders for which females are at greater risk. Using a fear conditioning and extinction protocol, they report that current *and past* COC users displayed greater fear returns in safety contexts. Their exploratory analyses linked fear regulation impairment to higher ethinyl estradiol (EE) doses and certain progestins.

Building on recent HC neuroscience reviews [1–3], we apply the dimensional hormone sensitivity lens [4] to contextualize these findings. We highlight robust methodological features while arguing that deeper consideration of HC characteristics—including progestin type, dose, and regimen—is key for clarifying why some individuals exhibit HC-related changes in fear regulation and other neurobehavioral risk processes.

## Hormone assays

One strength of the study is the use of liquid chromatography-tandem mass spectrometry to measure E2, EE, P4, and testosterone with specificity in saliva. Measurement of both endogenous (E2) and exogenous (EE) estrogens adds robustness; future studies should also measure exogenous progestins, endogenous P4, and affect-relevant metabolites like allopregnanolone (ALLO). Repeated hormone measures during HC use are critical, as fluctuations in endogenous and exogenous hormones can vary by route, formulation, and regimen. This could help clarify the most important hormonal mechanisms for understanding the impacts of HCs on affective outcomes (and avoid the incorrect assumption that endogenous levels remain stable in HC users).

## Dissecting COC formulation: type, dose, and regimen

The authors restricted the sample to individuals using COCs (containing EE + a progestin). This reduces route-related heterogeneity (i.e., excludes intrauterine, transdermal, subdermal methods), but COCs themselves remain highly diverse [1]. Below, we outline key sources of COC formulation variability that may interact with the findings [5] and individual differences in sensitivity [4].

## Progestin types

Although often treated as a single category, progestins differ markedly in their pharmacologic profiles, impact on ovarian steroid production (i.e., preventing ovulation), and associated neurobiological effects. As reviewed by Pletzer et al. [1], progestins differentially influence neurosteroids (e.g., ALLO) and neurotransmitters (e.g., GABA, serotonin) that are central to mechanisms of behavioral hormone sensitivity [4]. For instance, preclinical studies show that levonorgestrel decreases hippocampal ALLO, whereas drospirenone has a neutral effect. This distinction is clinically salient: paradoxical adverse responses to luteal ALLO rise underlie PMDD, a hormone sensitivity disorder for which drospirenone-containing COCs are uniquely efficacious and FDA-approved [4]. Progestins vary in receptor binding affinities (e.g., desogestrel > levonorgestrel at the glucocorticoid receptor), leading to diverse off-target effects to which individuals may be more or less sensitive [1]. These differences highlight that progestins should not be considered a homogeneous group, and variability in type may critically shape various hormone-related behavioral symptoms in susceptible individuals.

## Hormone dose

Davignon et al.’s comparison of EE dose across COCs is logical. However, comparisons between progestin doses are complicated, since higher-potency progestins are dosed at lower absolute levels. The authors’ “high” versus “low” progestin grouping by micrograms may conflate dose with type, since all participants in the anti-androgenic group (cyproterone acetate, drospirenone) are also in the “high-dose” progestin group (norethindrone acetate, cyproterone acetate, drospirenone). Additionally, exogenous hormone dose impacts endogenous steroid production, with higher doses leading to less follicular development and lower overall endogenous hormone production.

Unfortunately, there is no consensus method to compare differences in progestin doses and potencies. Some have suggested using “effective progestin activity” (dose multiplied by potency), while others created common scales based on dose required to inhibit ovulation. An alternative approach is needed to address “dose-dependent” progestin effects and avoid inappropriate binarization.

## Hormone regimen

COC regimen, referring to the schedule of active versus inactive pills (e.g., 21/7, 24/4, or continuous), is another underacknowledged source of variability. Although shortened hormone-free-intervals (HFI) of 24/4 achieve stronger ovarian suppression than 21/7, the HFI is associated with some follicular development (E2 surges) in both regimens relative to continuous use [3], thus determining the pattern of both exogenous and endogenous hormone exposure. Since studies of PMDD—which is characterized by surge-sensitive symptoms—demonstrate a delay of 1-2 weeks between surge and symptom peak, this introduces complexity in mapping hormone flux to symptoms [4].

A rare strength of this study’s design [5] was daily pill-use recording, allowing sensitivity analyses excluding those currently in the HFI. However, since hormone-surge-related symptoms are known to operate on a delay in susceptible individuals, more information is needed about effects of pill-pack transitions (e.g., *how many days since the HFI start? How many days since the end?*) and associated measurements of endogenous and exogenous hormone changes. Of note, triphasic regimens introduce additional weekly variability.

## Future directions

Ultimately, HC neuroscience will need experimentation and longitudinal designs to capture differences in hormone-sensitive symptoms during transition periods such as initiation, withdrawal, and around the HFI. Because people may be differentially sensitive to progestin types, doses, and regimens, as well as associated endogenous hormone fluctuations, head-to-head comparisons of formulations (e.g., comparing those with distinct effects on ALLO) offer an opportunity to elucidate key triggers of COC sensitivity. However, further neurobiological profile characterization of individual progestins is needed. This will allow us to better understand underlying mechanisms of their brain effects, enabling precision prescribing of COCs to minimize adverse effects and maximize benefits.
